# Identification and validation of a pyroptosis-related signature in identifying active tuberculosis via a deep learning algorithm

**DOI:** 10.3389/fcimb.2023.1273140

**Published:** 2023-11-01

**Authors:** Yuchen Liu, Lifan Zhang, Fengying Wu, Ye Liu, Yuanchun Li, Yan Chen

**Affiliations:** ^1^ Division of Infectious Diseases, Department of Internal Medicine, State Key Laboratory of Complex Severe and Rare Disease, Peking Union Medical College Hospital, Chinese Academy of Medical Sciences and Peking Union Medical College, Beijing, China; ^2^ Clinical Epidemiology Unit, Peking Union Medical College, International Clinical Epidemiology Network, Beijing, China; ^3^ Center for Tuberculosis Research, Chinese Academy of Medical Sciences and Peking Union Medical College, Beijing, China; ^4^ Peking Union Medical College, Chinese Academy of Medical Sciences and Peking Union Medical College, Beijing, China

**Keywords:** tuberculosis, latent tuberculosis infection, pyroptosis, bioinformatic, diagnostic model

## Abstract

**Introduction:**

Active tuberculosis (ATB), instigated by Mycobacterium tuberculosis (M.tb), rises as a primary instigator of morbidity and mortality within the realm of infectious illnesses. A significant portion of M.tb infections maintain an asymptomatic nature, recognizably termed as latent tuberculosis infections (LTBI). The complexities inherent to its diagnosis significantly hamper the initiatives aimed at its control and eventual eradication.

**Methodology:**

Utilizing the Gene Expression Omnibus (GEO), we procured two dedicated microarray datasets, labeled GSE39940 and GSE37250. The technique of weighted correlation network analysis was employed to discern the co-expression modules from the differentially expressed genes derived from the first dataset, GSE39940. Consequently, a pyroptosis-related module was garnered, facilitating the identification of a pyroptosis-related signature (PRS) diagnostic model through the application of a neural network algorithm. With the aid of Single Sample Gene Set Enrichment Analysis (ssGSEA), we further examined the immune cells engaged in the pyroptosis process in the context of active ATB. Lastly, dataset GSE37250 played a crucial role as a validating cohort, aimed at evaluating the diagnostic prowess of our model.

**Results:**

In executing the Weighted Gene Co-expression Network Analysis (WGCNA), a total of nine discrete co-expression modules were lucidly elucidated. Module 1 demonstrated a potent correlation with pyroptosis. A predictive diagnostic paradigm comprising three pyroptosis-related signatures, specifically AIM2, CASP8, and NAIP, was devised accordingly. The established PRS model exhibited outstanding accuracy across both cohorts, with the area under the curve (AUC) being respectively articulated as 0.946 and 0.787.

**Conclusion:**

The present research succeeded in identifying the pyroptosis-related signature within the pathogenetic framework of ATB. Furthermore, we developed a diagnostic model which exuded a remarkable potential for efficient and accurate diagnosis.

## Introduction

Infections caused by Mycobacterium tuberculosis (M.tb), can present as a dynamic spectrum, from latent tuberculosis infection (LTBI) to active tuberculosis (ATB). ATB dramatically impacts the morbidity and mortality rates, accounting for approximately 11.6% globally (Salari et al., 2023). A Despite the vast research focused on deciphering the pathogenic mechanisms underpinning LTBI and ATB, significant uncertainties persist (Alsayed and Gunosewoyo, 2023; Sengupta et al., 2023). The intricate interplay among M.tb, host immunological responses, and environmental factors potentially influences the diverse infection statuses of tuberculosis (Mohidem et al., 2021; Foreman et al., 2023; Yang et al., 2023). Additionally, the World Health Organization’s “End TB Strategy” signifies the critical need for notable advancements in tuberculosis diagnosis and therapies (WHO, 2022). However, the diagnosis of tuberculosis, especially extrapulmonary tuberculosis, has always faced many challenges. Smear microscopy is commonly applied technique to diagnose ATB, its high false negative rate made it one of the main reasons for the delay in case diagnosis. The classical gold standard for identifying ATB depends on culturing methods, which typically require more than two weeks (MacLean et al., 2020). Modern molecular screening methods such as Xpert have also been criticized for its high false negative rate (Engel et al., 2022). Interferon-Gamma Release Assays (IGRAs) can assist the diagnosis of ATB when etiological evidence is not available but performs poor when distinguishing ATB and LTBI (Chinese Society for Tuberculosis, Chinese Medical Association, 2022).

Pyroptosis, a proinflammatory form of programmed cell death, is triggered by gasdermin activation, further instigating an immediate immunological response against invasions (Ju et al., 2022). However, multiple studies indicate that pathogens may have advanced evasive measures that curtail pyroptosis, thereby enabling progressive infection (Chai et al., 2023). The role pyroptosis discharge in the genesis and progression of ATB, however, remains enigmatic.

The Back-Propagation (B-P) neural network, a prevalent deep learning neural network algorithm, has demonstrated substantial applicability (Wei et al., 2020). Incorporating machine learning techniques in biomedical research has facilitated the screening of novel biomarkers and the creation of more sophisticated diagnostic models (Chen and Wei, 2023; Gao et al., 2023; Jin et al., 2023). This methodological advancement appears promising in elucidating biomarkers critical for effective ATB management and diagnosis.

As the pathogenesis dictating the activation of tuberculosis remains complex and undiscovered, it is of imperative importance to elucidate the significance of pyroptosis in this context. This study endeavors to contribute to this overlooked area through a comprehensive bioinformatic analysis. The discovery from our research is anticipated to grant fresh perspectives into the understanding of tuberculosis.

## Methods

### Research design and data acquisition

Our investigation employed a comprehensive bioinformatics analysis with the aim of illuminating the Pyroptosis-Related Signature (PRS) in ATB. We identified differentially expressed genes (DEGs) within our exploratory cohort, subsequently utilizing weighted gene co-expression network analysis (WGCNA) to evaluate co-expressed genetic modules. This analysis was then followed by functional annotation to determine the module related to pyroptosis.

**Figure 1:**
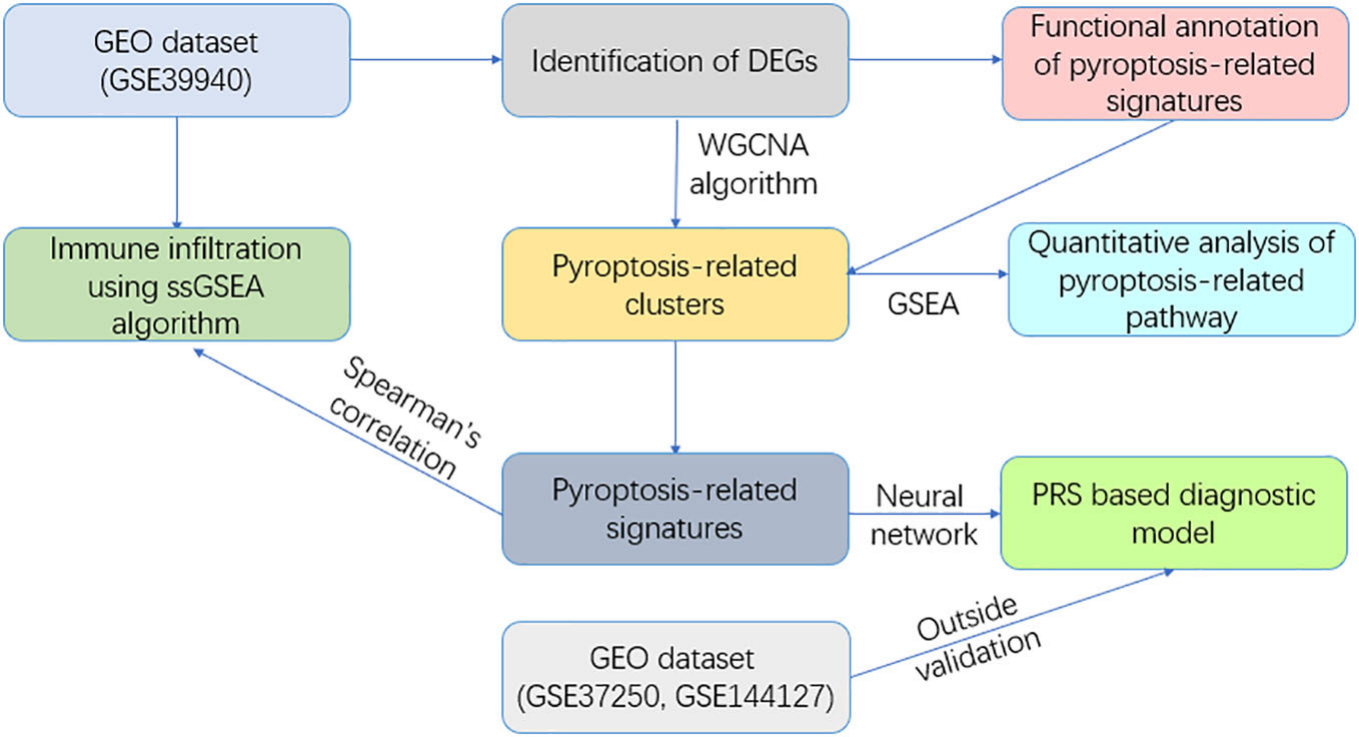
Flowchart of this study.

A neural network was employed to craft a diagnostic model, whose diagnostic prowess was verified within both the exploratory cohort and a separate validation cohort. In further pursuit of understanding the microenvironment linked to pyroptosis, a single-sample gene set enrichment analysis (ssGSEA) served to unearth the correlation between immune cells and pathways with our developed PRS model. The flowchart of the study is presented in **Figure 1**.

Transcriptomic and clinical informational data from patients containg ATB and LTBI were procured from the Gene Expression Omnibus (GEO) database (https://www.ncbi.nlm.nih.gov/geo/). We achieved our dataset by using search term “(‘active tuberculosis’ OR ‘ATB’ OR ‘active TB’)AND (‘LTBI’ OR ‘latent tuberculosis infection’)” and sorted by sample size, the top two datasets were selected as validation cohort 1 and exploratory cohort. The validation cohort is selected by the search term “(‘active tuberculosis’ OR ‘ATB’ OR ‘active TB’)AND ‘pneumonia’ AND ‘sarcoidosis’” and sorted by sample size. The exploratory cohort [GSE39940 (Anderson et al., 2014)] and the validation cohort [GSE37250 (Kaforou et al., 2013)] were both comprised of transcriptome information derived from peripheral blood samples, another validation cohort, GSE144127 (Hoang et al., 2021) compromising of 300 ATB samples, 61 pneumonia samples and 31 sarcoidosis samples is used to further validate the diagnostic potential of our model. Detailed clinical data pertaining to both cohorts is comprehensively summarized in **Table 1**.

**Table 1:** General information of the exploratory and the validation cohort.

	platform	Sample	ATB	LTBI
GSE39940	GPL10557	165	111	54
GSE37250	GPL10558	362	195	167

Module investigation and functional annotation

We first investigated the pyroptosis related genes through GO and KEGG analysis via ‘cluster profiler’ package in R (Yu et al., 2012). The expression of genes related to GO_BP pyroptosis, GO_BP regulation of cysteine-type endopeptidase activity involved in the apoptotic process and GO_BP regulation of cysteine-type endopeptidase activity involved in apoptotic process. The DEGs between ATB and LTBI patients of GSE39940 are analyzed through ‘limma’ package. DEGs were defined as |log2FC|>1 (FC, fold change) and adj.P<0.05.

Weighted Gene Co-expression Network Analysis (WGCNA) was utilized in our study to identify co-expressed genes in macrophages (Langfelder and Horvath, 2008). This method has the ability to convert co-expression correlation into connection weights or topological overlap values. The network type was kept as the “unsigned” type. Our WGCNA parameters were networkType =“unsigned”, minModuleSize = 20, mergeCutHeight = 0.25 and deepSplit = 2. The modules generated by WGCNA is shown in **Supplementary Material 1**.

We then used GSEA via ‘gsva’ package to quantize the difference of the defined module between the ATB and the LTBI patients (Hänzelmann et al., 2013; Powers et al., 2018). To investigate the role of pyroptosis, the enrichment of GO_BP pyroptosis, GO_BP regulation of cysteine-type endopeptidase activity involved in the apoptotic process and GO_BP regulation of cysteine-type endopeptidase activity involved in apoptotic process are illustrated through the ‘clusterProfiler’ of R (Yu et al., 2012). We selected the c5.all.v7.0.symbols.gmt gene set as the reference gene set.

### Development of diagnostic model using machine learning

We used B-P neural network algorithm to construct the diagnostic model, the ‘nnet’ package of R. The visualization is finalized via ‘Neural NetTools (Beck, 2018). A beanplot was leveraged to depict the risk affiliated with individuals within both the exploratory and validation cohorts.

### Assessment of the diagnostic potential of the PRS diagnostic model

We then examined the diagnostic potential of our diagnostic model in both cohorts by presenting the receiver operation curve (ROC). Its visualization is realized through ‘ROCR’ of R language. The area under the curve (AUC) is also calculated to demonstrate its diagnostic potential.

### Investigation of the immune infiltration

To scrutinize the associated microenvironment and immune infiltration, we performed ssGSEA to quantify the infiltration of 16 immune cells and 13 related immune pathways. The ‘GSVA’ package is used to perform ssGSEA in the GSE39940 (Hänzelmann et al., 2013).

### Statistical analysis

All statistical analyses were performed via R language (ver. 4.0.2) and R Studio. The Wilcoxon rank-sum test was used to compare non-normally distributed variables between groups. Spearman’s correlation test is used in discovering the relationship between immune infiltration and the PRS. A P<0.05 was considered statistically significant in the manuscript.

## Results

### Identification of pyroptosis-related genes and DEGs

**Figure 2:**
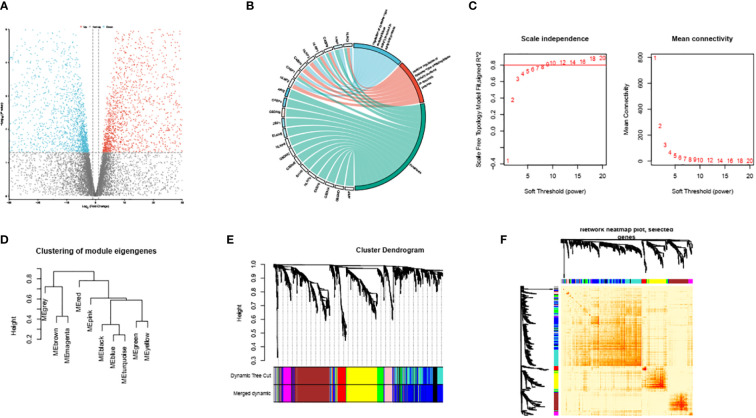
Volcano plots of partial DEGs between ATB and LTBI **(A)**. The identified PRS within GSE39940 **(B)**. The scale-free index and mean connectivity of various soft thresholds, the red line indicating the selected soft threshold **(C)**. Module dendrogram of the analyzed modules **(D)**. Cluster dendrogram demonstrating modules made up by genes with similar expression pattern **(E)**. Topological overlap matrix of the identified DEGs **(F)**.

To accomplish this task, we utilized the ‘GEOquery’ tool, a Bioconductor package that facilitates the downloading of gene expression data from the GEO (Gene Expression Omnibus) database. Specifically, we downloaded the gene expression matrix and related clinical data of the dataset GSE39940. The ‘limma’ package was used for data preprocessing. The DEG selecting criteria were set as following: log2|FC|≥1 and adj.P ≤ 0.05. (FC, fold change; adj.P: adjusted P value). We then identified 4103 down-regulated DEGs and 4934 up regulated DEGs. The DEGs are shown in **Figure 2A**.

### Construction and enrichment analysis of the modules

First, we performed GO analysis to identify the PRS in the down-regulated genes (**Figure 2B**). The PRS are overall suppressed in ATB compare to LTBI and are associated with GO_BP proptosis and GO_BP regulation of cysteine-type endopeptidase activity involved in apoptotic process. These intricate associations have been meticulously depicted and demonstrated in **Figure 2B**.

We conducted an unambiguous exploration of WGCNA in the DEGs, soft threshold is calculated by the ‘pickSoftThreshold’ function in the WGCNA. This soft threshold was eventually established at a value of 9, as represented in **Figure 2C**. Consequently, this led to the identification of the various encapsulating modules, the visual depiction of which can be observed in **Figure 2D**. Subsequently, we engaged in the construction of a hierarchical clustering tree. Each diverse branch of this tree epitomizes gene signatures exhibiting a trend of similarity within their expression and potential biological functionalities, as graphically presented in **Figure 2E**. For further consolidation of our findings, we proceeded to calculate the degree of connectivity between these identified modules. This process enabled us to study and understand the integral interactions that pervade such modules; **Figure 2F** elucidates this aspect of our research.

Following the initial phase of investigation, our accomplished task centered around discerning the module with the most intimate interactive connection to the process of pyroptosis. This was achieved through an in-depth deployment of descriptive GO analysis, the result of which culminated in us identifying this attribute within module 1.

Upon this recognition, we further advanced to utilizing GSEA to quantitatively assess and thereby determine the extent of activity associated within relative biological pathways, explicitly situated within module 1. This analytical method was paramount, serving as a facilitative tool to substantiate our investigation with tangible numeric values.

Furthermore, we meticulously identified the distinct PRS within module 1, specifically pinpointing the presence of AIM2, CASP8, and NAIP). These are vital pieces of information since they could provide indicators to the understanding of the dynamics underlying this process.

**Figure 3:**
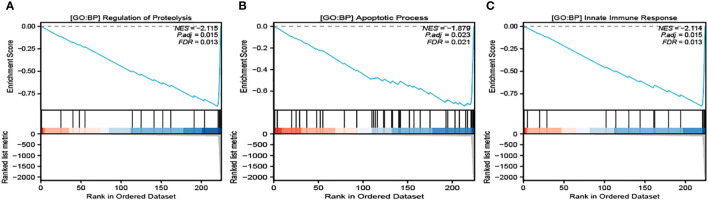
GSEA analysis of the related pathways in module 1. GO : BP Regulation of Proteolysis **(A)**, GO : BP Apoptosis Process **(B)** and GO : BP Innate Immune Response **(C)** are significantly enriched in module 2.

It’s noteworthy to clarify that our selected module demonstrated the most intimate connection with three specific Gene Ontology Biological Processes (GO : BP), all of which are crucial to the process of cellular death and immune response. These identified pathways are Regulation of Proteolysis (**Figure 3A**), Apoptosis Process (**Figure 3B**), and Innate Immune Response (**Figure 3C**). These identified closeness of relationships provide an in-depth understanding of the molecular interactions and regulatory functions taking place within the process of pyroptosis.

### Construction and evaluation of the PRS model

**Figure 4:**
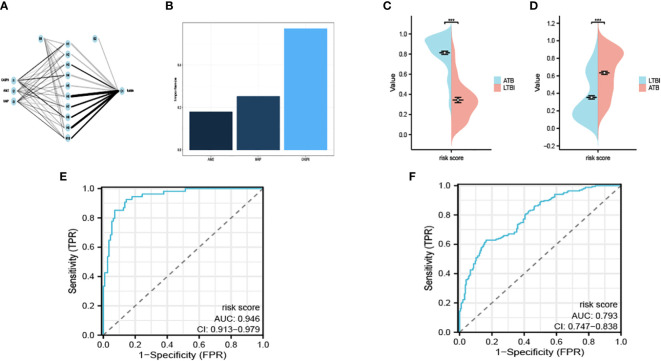
Interaction of the hidden layer, the input layer and the output layer **(A)**. The importance of each input variable in the model **(B)**. The risk score calculated in the exploratory **(C)** and the validation cohort **(D)**. The ROC of GSE39940 **(E)** and ROC37250 **(F)**. ***P < 0.05.

We acquired the expression data of AIM2, CASP8 and NAIP from GSE39940 as the training set. B-P neural network algorithm, a robust method for predictive model construction due to its ability to better adjust internal parameters through iterative comparison of original and predicted outcomes, was crucial in effectuating the development of the diagnostic model under consideration (**Figure 4A**). The model is demonstrated in **Supplementary Material 1**. In order to ascertain the significance and influence of individual predictors on the output of our model, we utilized the ‘garson’ algorithm, instituted within the ‘nnet’ package of R programming language – an esteemed tool for statistical analysis which enables an insightful comprehension of complex patterns in our data (**Figure 4B**).

We remained committed to unravelling the undiscovered diagnostic potential of our meticulously crafted model. We obtained the expression of AIM2, CASP8 and NAIP from GSE37250 as an outside validation cohort. We calculated the risk score determined by our model. The calculated risk scores were lucidly exhibited through the deployment of bean plots, visually representing the distribution of risk scores in our exploratory cohort (**Figure 4C**) and the validation cohort (**Figure 4D**). Further, we opted to present the ROC of GSE39940 is shown in **Figure 4E** and the ROC of GSE37250 is shown in **Figure 4F**. The AUC of GSE39940 and GSE37250 is 0.946 and 0.793, these results communicate an excellent diagnostic potential housed by our model, hence holding promise for advancing diagnostic processes in the field.

**Figure 5:**
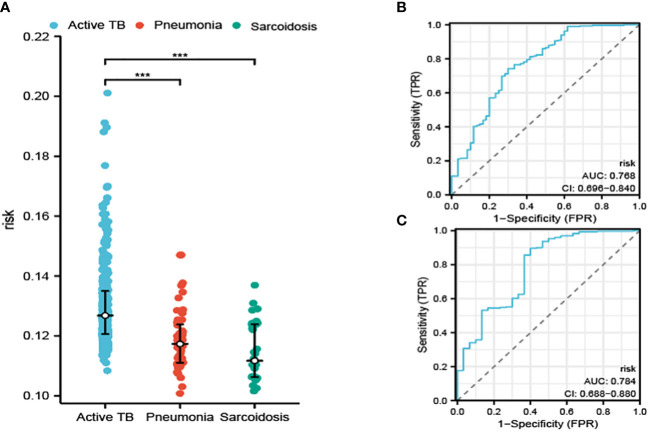
The risk determined by our model in distinguishing ATB from pneumonia and sarcoidosis **(A)**. The ROC curve for distinguishing ATB from pneumonia **(B)** and sarcoidosis **(C)**. ***P<0.001.

In addition to our initial findings, we also embarked on an exploration of the diagnostic capabilities of our constructed model in terms of its potential to differentiate between ATB, pneumonia, and sarcoidosis, an investigation which utilized an entirely separate dataset as portrayed in **Figure 5A**. The performance of the diagnostic procedure was further assessed and validated by the creation of ROC curves, graphically represented in **Figures 5B, C**. These provided a visualization of the dichotomy between sensitivity and specificity, thereby reflecting the overall diagnostic accuracy of our model.

The AUC was calculated for our model. These calculations resulted in the AUC values of 0.768 and 0.784, which affirm the potential of our model to produce reliable results while distinguishing between ATB, pneumonia, and sarcoidosis. The values solidified our confidence in the model’s clinical utility.

### Analysis of the microenvironment and related genes

**Figure 6:**
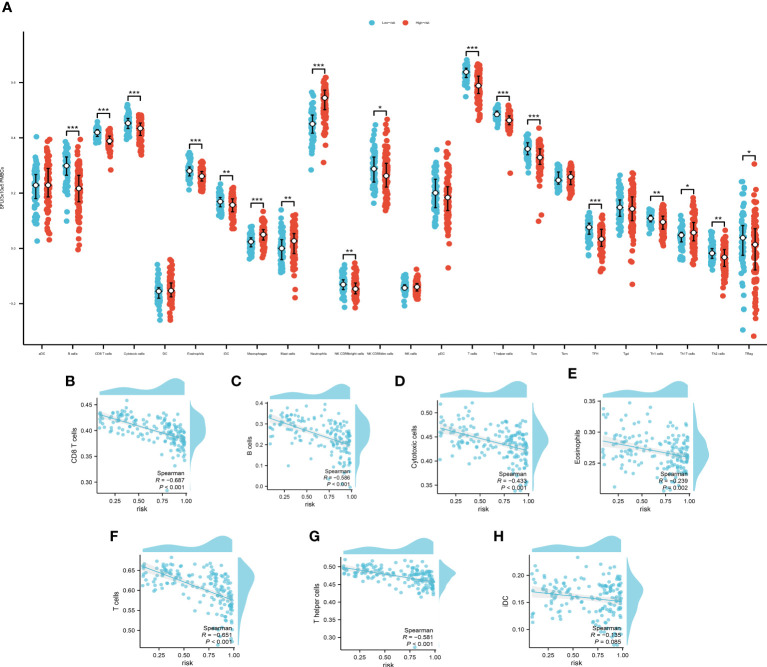
**(A)** The immune infiltration of the high- and low- risk group, *P<0.05, **P<0.01; ***P<0.001. Spearman’s correlation analysis of B cells **(B)**, CD8+ T cells **(C)**, cytotoxic cells **(D)**, eosinophils **(E)**, T cells **(F)**, T helper cells **(G)** and iDCs **(H)**.

We used ssgsea to analyze the immune infiltration associated with pyroptosis (**Figure 6A**).

The cut off value of risk score is determined by the specificity and sensitivity determined through the ROC and is settled as 0.64725. Among which, B cells, CD8+ T cells, cytotoxic cells, eosinophils, T cells, T helper cells and immature Dendritic Cells (iDCs) displayed a noteworthy disparity when comparing the high-risk group with the low-risk counterparts. These findings not only contribute additional granular insights but also pave the way for further explorations of potential mechanisms underpinning disease progression. We then used Spearman’s correlation to examine the relationship of the immune cells with our model. The Spearman’s correlation between B cells (**Figure 6B**), CD8+ T cells (**Figure 6C**), cytotoxic cells (**Figure 6D**), eosinophils (**Figure 6E**), T cells (**Figure 6F**), T helper cells (**Figure 6G**) and iDCs (**Figure 6H**) with the PRS are evaluated and demonstrated. The B cells, CD8+ T cells, T cells and T helper cells showed a significant correlation with the PRS (|R|>0.5, P<0.01). These findings underscore the potential of these cellular subpopulations in playing a pivotal role in disease progression as reflected by our model’s risk prediction.

### Investigation of related PRS genes

**Figure 7:**
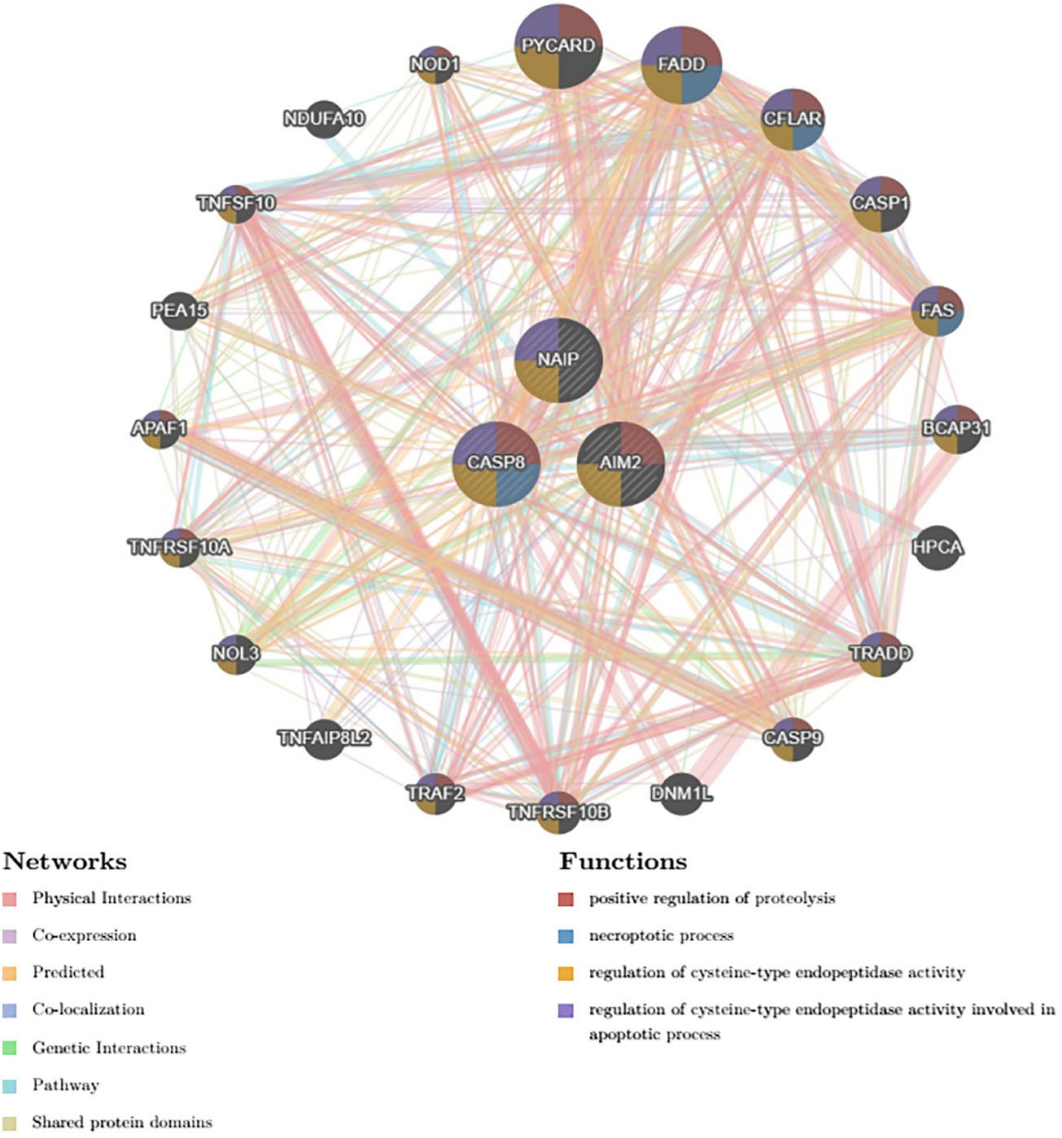
Top 20 genes that shares closest relationship with the PRS.

We employed the use of GeneMANIA, an advanced gene-centric data-mining tool accessible at http://GeneMANIA.org. Our primary objective was to delve deeper into the intricate genetic interdependencies, specifically focusing on identifying the twenty most intimately correlated genes in relation to the Polygenic Risk Score (PRS), the details of which have been visually elucidated in **Figure 7**.

In engaging with this complex network of genes, our analysis precipitated an understanding of several predicted associated biochemical pathways. Notably, these included the positive regulation of proteolysis, denoting a mechanism responsible for the induction of the breakdown of proteins into smaller polypeptides or single amino acids.

Furthermore, the necrotic processes emerged as a significant conjoined element acting within the network—these are pathways leading to programmed cell death, particularly of a pathological nature, where premature death of cells in living tissue is caused by factors external to the cells or tissues themselves.

The investigation also highlighted the regulation of cysteine-type endopeptidase activity—suggesting an involvement of enzymes that use a cysteine residue in their active site and function in the regulation of diverse cellular processes through protein hydrolysis.

Lastly, the analysis brought to light the role of cysteine-type endopeptidase activity implicated in the apoptotic process, thus providing insights into the programmed cell death mediated by the targeted activation of such enzymes for cellular self-destruction.

These predicted associated pathways underscore the intricate interplay of genetic and biochemical interactions inextricably entwined with the PRS investigated, and are instrumental in deepening our understanding of the topics at hand.

## Discussion

Tuberculosis, a profoundly pervasive infectious disease, continually affects vast multitudes of individuals globally, recording overwhelming figures that run into the millions (Ding et al., 2022). Although a large proportion of individuals infected by M.tb conspicuously exhibit no symptoms (Gong and Wu, 2021). There remains a subpopulation of individuals where the disease manifests itself in an array of clinical presentations. ATB is known for its clinical heterogeneity, comprise pulmonary or systemic symptoms such as febrile conditions, a persistent cough, excruciating chest discomfort, and unintentional weight loss; such individuals are characteristically diagnosed as patients with ATB (Furin et al., 2019). The current golden diagnostic criterion relies on culture- and sputum- based technology, which have been criticized for its low sensitivity (Kontsevaya et al., 2023). The interferon-γ release assay (IGRA) and TST, have been used to screen for tuberculosis infection, however, such tests are unable to distinguish LTBI and ATB (Ludi et al., 2023). By elucidating the complex biological processes and pathways that trigger the transition of tuberculosis from a latent state to an active disease, we not only enhance our understanding of tuberculosis pathogenesis but also unfold a potential diagnostic technique.

The pathogenesis that precipitates the transition from latent infection to active disease, colloquially known as tuberculosis activation, remains a formidable scientific enigma of considerable proportions. Concurrently, an intriguing focus has been directed towards the potential role played by programmed cell death mechanisms within this complex disease process. The functions of various forms of programmed cell death such as apoptosis – a controlled cellular suicide, and pyroptosis – a highly inflammatory form of programmed cell death, are under rigorous investigation in relation to their specific contributions towards the advancement and progression of tuberculosis (Alemán, 2015; Lam et al., 2017).

Pyroptosis, a programmed cell death that considered to be part of the innate immune response in host defense while facing pathogens (Brokatzky and Mostowy, 2022). Studies have identified a phospholipid phosphatase produced by M.tb that inhibits the host inflammasome pyroptosis pathway, PtpB (Chai et al., 2022). The implications of these findings suggest that the evaluation of pyroptosis in patients with ATB and those with latent tuberculosis infection (LTBI) could hold immense potential for the early detection of the former. Furthermore, the potential to target pyroptosis emerges as an innovative paradigm shift that could offer new avenues for therapeutic intervention, particularly in dealing with the persistent problem of drug-resistant TB.

Within the context of our comprehensive scientific investigation, our primary objective was to shed light on a genetic signature pertaining to pyroptosis, with the potential to be leveraged as a viable diagnostic tool for ATB. In order to accomplish this endeavor, our initial step was to carry out a comparative analysis of transcriptomic data sourced from ATB and LTBI patients. This analytical process yielded 4103 down-regulated DEGs and 4934 up-regulated DEGs. Following this, we implemented the WGCNA to identify the co-expressed modules. Further, we utilized GO enrichment analysis to distinguish the module share the closest interaction with pyroptosis and identified the 3 PRS: AIM2, CASP8 and NAIP. Moreover, we applied GSEA to quantify the related pathway of pyroptosis. Next, we used B-P neural network to construct a PRS model. It demonstrated excellent diagnostic potential distinguishing ATB from LTBI patients in both the exploratory and an outside validation cohort. We used the developed PRS model to divide the cohort into a high- and a low-risk group, followed by ssgsea to illustrate the immune infiltration. Furthermore, we analyzed the related genes and their predicted pathways that are closely related to the PRS.

The PRS model elucidated based on neural network of AIM2, CASP8 and NAIP. AIM2 (absent in melanoma 2) is a pivotal component of the AIM2 inflammasome, well-documented for its ability to perceive double stranded DNA (Lee et al., 2021). has observed the critical functionalities of inflammasomes, specifically in promoting cell death in cells laden with pathogens (Man et al., 2017). Substantiation for this can be found in studies on AIM2-deficient models, revealing enhanced susceptibility to intratracheal infection with Mycobacterium tuberculosis (Saiga et al., 2012). CASP8(cysteine-aspartic acid protease 8) is a constituent member of the caspase family, acknowledged for its involvement in the sequence leading to pyroptosis. Our investigation uncovered scientific reports indicating that the presence of extracellular RNA fragments from Mycobacterium tuberculosis sparks increased expression of CASP8 in *in-vitro* environments (Fritsch et al., 2019; Zhang et al., 2021). NAIP(NLR family apoptosis inhibitory protein) collaborates with NLRC4 to form the NAIP-NLRC4 inflammasome (Barnett et al., 2023). Although its involvement in ATB have not been reported, *in vivo* experiments highlight the significance of its involvement in infections festering in the lung, spleen, liver, and systemic sepsis-like conditions (Bauer and Rauch, 2020).

Our comprehensive study of the intertwined web of bioinformatics slated an objective to unravel the signatures associated with pyroptosis in patients afflicted with tuberculosis. This investigative venture has shown us the potential of a diagnostic model, bolstered by a machine learning mechanism. We verified the diagnostic capacities of the model using an independent cohort. We have also verified our model in a 3rd cohort to distinguish between ATB and pneumonia/sarcoidosis, which reaffirmed its usefulness in a clinical context, confirming its potential for clinical assessment and management. Our finding might serve as resource for clinicians to improve the patients’condition through early diagnosis and targeted medical strategy.

Though our research presented promising results, several limitations exist. Firstly, the sample size of our study is rather limited, therefore it is crucial to assess our model in larger population. Secondly, our study lacks verification from *in vitro*/*in vivo* experiments, in which case the authors manage to use independent cohorts to assess the diagnostic potential of our model, to ensure the strength of our result. Future researchers could focus on evaluating the underlying mechanism of the signatures involved in our model through laboratory experiments, including validating the differential expression of candidate genes *in vitro* or using animal models to investigate the relevance of these genes in ATB pathogenesis.

In summation, our precarious yet promising research undertakings continue to unravel the role of pyroptosis in ATB and bring to light the potential of PRS as an invaluable tool in diagnosing ATB. Nonetheless, for a comprehensive understanding and evaluation of the clinical utility of our proposed approach in diagnosing and management of ATB, substantial expansions in the future research are fundamentally imperative.

## Data availability statement

The datasets presented in this study can be found in online repositories. The names of the repository/repositories and accession number(s) can be found below: https://www.ncbi.nlm.nih.gov/, GSE37250 https://www.ncbi.nlm.nih.gov/, GSE39940.

## Ethics statement

Ethical approval was not required for the study involving humans in accordance with the local legislation and institutional requirements. Written informed consent to participate in this study was not required from the participants or the participants’ legal guardians/next of kin in accordance with the national legislation and the institutional requirements.

## Author contributions

YucL: Conceptualization, Data curation, Formal Analysis, Investigation, Methodology, Project administration, Software, Validation, Visualization, Writing – original draft, Writing – review & editing. LZ: Conceptualization, Formal Analysis, Funding acquisition, Methodology, Project administration, Resources, Supervision, Visualization, Writing – review & editing. FW: Conceptualization, Data curation, Formal Analysis, Funding acquisition, Writing – original draft, Writing – review & editing. YeL: Data curation, Formal Analysis, Funding acquisition, Visualization, Writing – original draft. YuaL: Data curation, Formal Analysis, Investigation, Resources, Software, Writing – original draft. YC: Conceptualization, Formal Analysis, Software, Validation, Visualization, Writing – review & editing.
