# Supplementary material for: Identification and validation of a pyroptosis-related signature in identifying active tuberculosis via a deep learning algorithm
Source: Front Cell Infect Microbiol. 2023 Nov 1;13:1273140. doi: 10.3389/fcimb.2023.1273140 (PMC10646574; doi:10.3389/fcimb.2023.1273140)
Supplement: Supplementary file 1 [file Table_1.docx]

a 3-10-1 network with 51 weights

options were - decay=0.12

b->h1 i1->h1 i2->h1 i3->h1

0.03 0.47 0.09 -0.28

b->h2 i1->h2 i2->h2 i3->h2

0.01 -0.22 0.04 0.16

b->h3 i1->h3 i2->h3 i3->h3

-0.02 -0.49 0.01 0.24

b->h4 i1->h4 i2->h4 i3->h4

0.00 0.40 -0.09 0.23

b->h5 i1->h5 i2->h5 i3->h5

0.00 0.00 0.01 0.00

b->h6 i1->h6 i2->h6 i3->h6

-2.17 0.00 0.00 0.00

b->h7 i1->h7 i2->h7 i3->h7

-0.03 -0.66 0.23 -0.38

b->h8 i1->h8 i2->h8 i3->h8

-0.05 0.49 -0.10 0.07

b->h9 i1->h9 i2->h9 i3->h9

-1.91 0.01 0.00 0.00

b->h10 i1->h10 i2->h10 i3->h10

0.02 -0.65 0.13 0.27

b->o h1->o h2->o h3->o h4->o h5->o h6->o h7->o h8->o h9->o h10->o

-0.74 -1.62 -0.84 0.70 -1.37 -0.75 2.51 1.13 1.27 2.21 1.23
